# Disseminated subcutaneous sporotrichosis in an immunocompetent patient: A case report and a systematic literature review

**DOI:** 10.1016/j.mmcr.2025.100739

**Published:** 2025-09-26

**Authors:** Guangqi Zhu, Luwen Zhang, Yan Wang, Zheen Zhang

**Affiliations:** a Department of Infectious Diseases, Nanjing Drum Tower Hospital, Affiliated Hospital of Medical School, Nanjing University, Nanjing, Jiangsu, China; b Institute of Viruses and Infectious Diseases, Nanjing University, Nanjing, Jiangsu, China; c Department of General Medicine, Zhanmao Community Health Service Center, Zhoushan, 316021, China; d Department of Infectious Disease, Zhoushan Hospital, Wenzhou Medical University, Zhoushan, 316021, China

**Keywords:** *Sporothrix schenckii*, disseminated subcutaneous sporotrichosis, Immunocompetent

## Abstract

Sporotrichosis is a subacute and chronic infectious disease caused by the infection of the skin, subcutaneous tissues, and nearby lymphatic vessels by the sporotrichosis complex. In this study, we report an immunocompetent case of disseminated subcutaneous sporotrichosis and provide a literature review of all case reports of disseminated subcutaneous sporotrichosis for which species identification was performed. A 74-year-old male patient with normal immune function was diagnosed with disseminated subcutaneous sporotrichosis. Sporadic red masses of varying sizes with local ulceration were observed on his right forearm, wrist, and little finger. Pathology of the mass in the right upper extremity indicated chronic suppurative inflammation of the subcutaneous soft tissue. The mNGS results indicated the presence of *Sporothrix schenckii*. Following the standard treatment with itraconazole, the patient's skin lesions healed without scarring or pigmentation. Disseminated subcutaneous sporotrichosis is less common in patients with a normal immune function. mNGS testing aids in the clear diagnosis of sporotrichosis and guides antifungal drug selection during treatment.

## 1 Introduction

Sporotrichosis is a subacute and chronic infectious disease caused by infection of the skin, subcutaneous tissues, and nearby lymphatic vessels by the sporotrichosis complex [1]. The sporotrichosis complex includes *Sporothrix schenckii*, *Sporothrix brasiliensis*, *Sporothrix globosa*, *Sporothrix mexicana*, *Sporothrix lueri*, and *Sporothrix pallidum* [2]. Its skin lesions are mainly characterized by ulcers, abscesses, subcutaneous nodules, papules, and erythema, which often involve the unilateral upper and lower extremities, face, and may also spread to the gastrointestinal tract, bones and joints, lungs, eyes, and central nervous system. Immunocompromised or immunodeficient individuals are more susceptible to hematogenous dissemination to all organs of the body [3]. Histopathology frequently shows granulomas, most commonly pyogenic granulomas [4].

Several studies have found [5,6] that different clinical types of sporotrichosis infections are associated with the immune status of the organism, virulence of the sporotrichosis, and genetic differences. Zhang et al. [7] found that genotypic and virulence variants of strains may contribute to the emergence of a cutaneous disseminated form of sporotrichosis. However, no study has clearly revealed whether there is a correlation between different Sporothrix species and clinical types. In this study, we report an immunocompetent case of disseminated subcutaneous sporotrichosis and provide a literature review of all case reports of disseminated subcutaneous sporotrichosis for which species identification was performed.

## 2 Case presentation

On May 15, 2021, a 74-year-old male patient visited the infection department of our hospital for treatment, complaining of a right forearm lump caused by a 2-month hand injury for half a month. Two months prior, the patient had been stabbed by a tree branch in the mountains and had hurt his right little finger. The patient experienced little pain and tried to pick out the tree branch without success, and then went to the local hospital for debridement treatment. The patient developed a red rash on the right forearm half a month ago, which gradually expanded into a painless red mass six days later and gradually spread from the right forearm to the right forearm. After treatment in a local hospital with levofloxacin, clindamycin, and sulfamethoxazole intravenous anti-infective therapy 10 days later, the mass on the right forearm of the patient did not improve significantly.

**Fig. 1:**
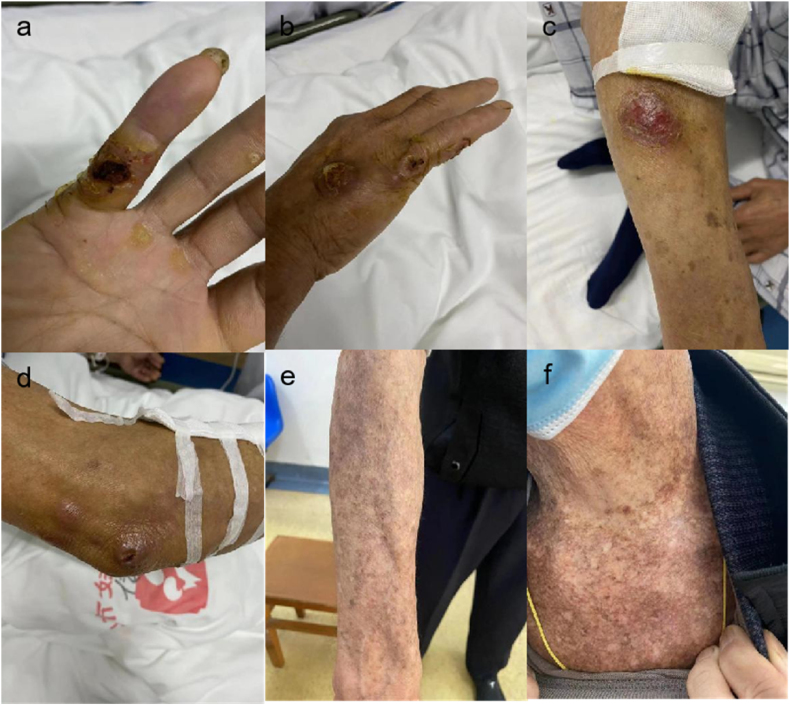
Appearance of skin lesions on the patient's fingers, back of the hand, arms, and neck.

**Fig. 2:**
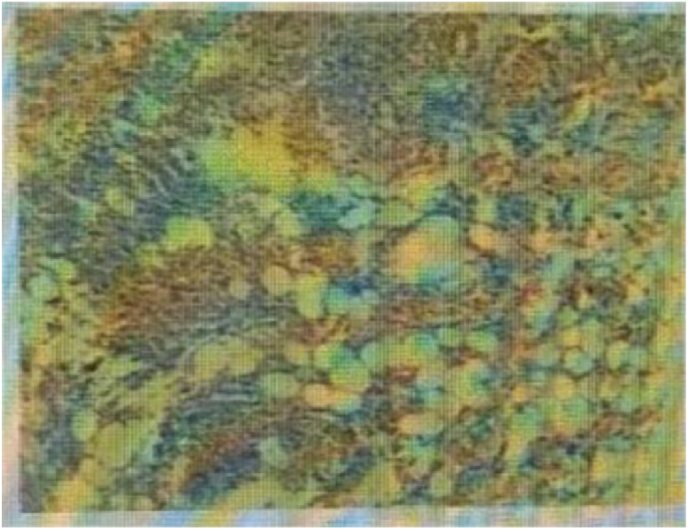
Pathological examination of biopsy tissue at the right arm nodule of the patient shows chronic suppurative inflammation of subcutaneous soft tissue.

He had a history of gout for 4 years and took painkillers when he had an attack. Physical examination revealed a sporadic red mass in the right forearm, wrist, and little finger, varying in size, and local ulceration (Fig. 1). Auxiliary examination revealed a white blood cell count of 10.5 × 10^9^/L. A right upper arm MRI scan showed an effusion in the subcutaneous fat layer of the forearm and the lower part of the upper arm, as well as subcutaneous swelling in the right little finger. Pathology of the mass in the right upper extremity indicated chronic suppurative inflammation of the subcutaneous soft tissue (Fig. 2). We administered fluconazole and sodium chloride injection (0.4 g qd) via intravenous drip, and sulfamethoxazole 2 tablets tid orally as empirical treatment.

The mNGS results indicated the presence of *Sporothrix schenckii*. Based on the skin lesions observed on the patient's right finger, dorsal hand, arm, and neck, with no evidence of involvement of other organs, we combined the clinical, pathological, and mNGS test results to ultimately diagnose the patient with disseminated sporotrichosis caused by *S. schenckii*. According to the diagnosis, the treatment regimen was changed to Itraconazole capsules at 0.2 g qd po. During the treatment process, the patient's mass oozed pus, leading to a change in the dosage to Itraconazole capsules 0.2 g bid po. After 2 weeks of treatment, the lesions on the right upper limb dried up and were significantly reduced in size compared to before treatment, without redness, oozing, or new lesions. The patient was discharged and continued oral itraconazole capsules for consolidation therapy. Follow-up 4 months after discharge showed that the skin lesions had healed with no scarring or pigmentation.

## 3 Discussion

Sporotrichosis is usually divided into cutaneous and extracutaneous forms, according to clinical typing, and the cutaneous form can be further divided into fixed, lymphovascular, and cutaneous disseminated forms. The cutaneous disseminated form is relatively rare and has a variety of lesion patterns, including ulcerated nodules and warty plaques, crusts, erythematous scales, papules, and pustules [8,9]. It usually occurs in immunocompromised patients with HIV or medically immunosuppressed patients, in addition to impaired cellular immunity, alcoholism, and diabetes mellitus, which are also considered risk factors for disseminated disease [[10], [11], [12]].

We conducted a systematic literature search on cutaneous disseminated cases identified by species in September 2025 using PubMed, Scopus, and Web of Science with the following keywords: ("sporotrichosis") and ("skin disseminated"). We further screened and removed the articles that did not undergo species identification. A total of 16 out of 22 cases of disseminated subcutaneous sporotrichosis caused by different strains of the *Sporothrix* sp. complex were identified through strain identification, as shown in Table 1.

**Table 1:** Cases of patients with disseminated subcutaneous sporotrichosis identified by species as of September 2025 in literature search reports.

Year	Nation	Age(years)	Gender	Medical illnesses	Medication	Outcome	Sporothrix species	Diagnostic method	Reference
2022	Canada	52	Male	None	Itraconazole	Cure	*Sporothrix schenckii*	PCR	Telles et al. [13]
2022	Japan	76	Male	IgG4 related diseases	Potassium iodide、itraconazole and terbinafine	Cure	*Sporothrix globosa*	PCR	Nomoto et al. [14]
2022	China	55	Female	Tuberculous peritonitis	Itraconazole	During follow-up	*Sporothrix globosa*	PCR	Shi et al. [15]
2021	Mexico	21	Male	None	Potassium iodide	Cure	*Sporothrix schenckii*	PCR	Herrera et al. [16]
2021	America	44	Female	After surgery for breast ductal carcinoma in situ	Amphotericin B、itraconazole and terbinafine	Recurrence	*Sporothrix schenckii*	PCR	Zambrano et al. [17]
2021	Brazil	41	Female	Renal transplant	Amphotericin B、itraconazole and terbinafine	Cure	*Sporothrix brasiliensis*	PCR	Fichman et al. [18]
2021	Brazil	43	Male	Renal transplant	Amphotericin B、itraconazole and terbinafine	Death	*Sporothrix brasiliensis*	PCR	
2020	Mexico	45	Male	Inflammatory bowel disease	Itraconazole	Cure	*Sporothrix schenckii*	PCR	Rivero et al. [19]
2020	Brazil	38	Female	HIV	Amphotericin B	Cure	*Sporothrix brasiliensis*	PCR	Poester et al. [20]
2020	Brazil	61	Female	None	Itraconazole、amphotericin B and potassium iodide	Recurrence	*Sporothrix brasiliensis*	PCR	Valeriano et al. [21]
2020	Brazil	26	Female	None	Itraconazole	Cure	*Sporothrix brasiliensis*	PCR	
2020	Brazil	64	Male	None	Itraconazole	Cure	*Sporothrix brasiliensis*	PCR	
2020	Brazil	46	Female	None	Itraconazole	Cure	*Sporothrix brasiliensis*	PCR	
2019	America	62	Male	Iatrogenic immune suppression	Terbinafine	Cure	*Sporothrix schenckii*	PCR	White et al. [10]
2018	Brazil	13	Female	None	Itraconazole	Cure	*Sporothrix brasiliensis*	DNA sequencing	Fernandes et al. [22]
2017	Brazil	35	Male	HIV with severe immune suppression	Amphotericin B and itraconazole	Cure	*Sporothrix brasiliensis*	PCR and DNA sequencing	Biancardi et al. [23]
2017	Brazil	25	Male	HIV with severe immune suppression	Amphotericin B and itraconazole	Cure	*Sporothrix brasiliensis*	PCR and DNA sequencing	
2017	Brazil	43	Male	HIV with severe immune suppression	Amphotericin B and itraconazole	Cure	*Sporothrix brasiliensis*	PCR and DNA sequencing	
2015	Brazil	61	Male	None	Terbinafine	Recurrence	*Sporothrix brasiliensis*	PCR	Freitas et al. [24]
2012	Canada	44	Male	Leukemia	Amphotericin B、itraconazole and posaconazole	Recurrence	*Sporothrix schenckii*	DNA sequencing	Bunce et al. [25]
2011	Mexico	36	Male	None	Itraconazole、amphotericin B and potassium iodide	Recurrence	*Sporothrix schenckii*	Molecular biology methods	Cabello et al. [26]
2020	China	59	Female	Diabetes	Itraconazole	Cure	*Sporothrix globosa*	DNA sequencing	Xia et al. [27]

The patients included in the review were identified by molecular characterization of the strain type, including 31.8 %(7/22) of *Sporothrix schenckii*, 54.5 %(12/22) of *S. brasiliensis*, and 13.6 %(3/22) of *S. globosa*. Among them, 8 patients were in a state of immune suppression, including the use of immunosuppressant medications and the presence of HIV. The clinical manifestations were mostly erythema ulcers, papules, nodular and warty plaques, and purulent secretions in the limbs and body. Itraconazole, amphotericin B, and potassium iodide are the most commonly used drugs. Among these patients, 15 were cured, 5 experienced recurrence, 1 died, and 1 was still under follow-up.

These patients had the highest number of *Sporothrix schenckii* cases, which is consistent with the results of Zhuang et al. [28], suggesting that the possibility of the predominance of *Sporothrix schenckii* in disseminated subcutaneous sporotrichosis cannot be excluded, but further basic research is needed to clarify the mechanism and clinical validation of large samples. The distribution of these cases, mainly in Mexico, Brazil, the United States, Canada, and China, is characterized by a distinct geographic region, whereas *Sporothrix schenckii* detected in this study is relatively rare in China. Since most of the cases reported so far in China are spherical sporotrichosis with weak virulence, most show a fixed or lymphovascular type, and very few cases report a skin-disseminated type, which can be easily confused with diseases such as gangrenous pyoderma [29]. Therefore, when patients with disseminated multiple ulcerative nodules on the skin are seen in countries outside of North America, such as China, clinicians should consider the occurrence of this condition during clinical consultation.

There is a correlation between the severity of sporotrichosis and virulence of Sporothrix, with significant differences in drug resistance and virulence among different species of strains. Marimon et al. [30] found in vitro testing of antifungal drugs showed that *Sporothrix mexicana* was the most resistant, whereas *Sporothrix brasiliensis* had the best response to the action of antifungal drugs. Rodrigo et al. [31] found that different sporotrichous filamentous strains may be associated with different clinical manifestations and therapeutic responses, and that patients with *Sporothrix schenckii* had significantly longer treatment times than those with *Sporothrix brasiliensis*, which is consistent with *Sporothrix brasiliensis* being more sensitive to antifungal drugs than *S. schenckii*. In terms of virulence, the results of a comparative study on the pathogenicity of different sporotrichous fungi using an immunologically active mouse model showed that *Sporothrix brasiliensis* was the strongest, *Sporothrix schenckii* was stronger, and *Sporothrix globosa* and *Sporothrix mexicana* were less virulent in the mouse model [32]. Emma et al. [33] included strains from several regions of Venezuela for species identification from 1973 to 2013. It was found that all spherical sporotrichums in the study showed fixed sporotrichosis, which may be related to the low virulence of the strain, whereas all lymphovascular cases and the only case of cutaneous dissemination, as well as 33 % of the fixed cases, were *Sporothrix schenckii*. In conclusion, the high virulence of *Sporothrix schenckii*, second only to *Sporothrix brasiliensis*, and its insensitivity to antifungal drugs make patients with *Sporothrix schenckii* infections in the early stages of the disease more susceptible to cutaneous dissemination, which may be the reason why disseminated subcutaneous sporotrichosis is most commonly associated with *Sporothrix schenckii* infections.

The cutaneous disseminated form is common in immunodeficient patients but rare in immunocompetent patients. In recent years, several cases have been reported in immunocompetent patients presenting with a cutaneous disseminated phenotype, but the pathogenic mechanism is not clear, and these studies were considered atypical. However, Zhang et al. [34] suggested that the different clinical manifestations of sporotrichosis may be related to genotypic and virulence variants of the strains, but not to the effect of the host immune status, for which further studies are still needed. In this study, the patient was immunologically normal but also showed cutaneous dissemination on the right forearm, wrist, and little finger, which may be due to the patient's age and the lack of proper antifungal treatment in the early stages of the disease; therefore, the disease progressed in a delayed manner, leading to the appearance of the cutaneous disseminated form.

According to the updated 2007 Practice Guidelines for the Management of Sporotrichosis in the United States [35], itraconazole 100–200 mg/d is recommended for patients with limited access to the skin, subcutaneous tissues, and some lymphatic vessels, and fluconazole 400 mg/day is used if it is intolerable. The 2016 Guidelines for the Diagnosis and Treatment of Sporotrichosis in China [36] recommend itraconazole as the treatment of choice, with a dose of 200–400 mg/d orally for adults and 5 mg/kg/d orally for children for a course of 3–6 months or longer. Potassium iodide, which is the standard treatment for sporotrichosis, has adverse effects on salivary gland enlargement and rash, and is no longer recommended as the first choice. The treatment regimen for sporotrichosis includes topical measures (thermotherapy), polyenes (amphotericin B), and allylamines (terbinafine). Amphotericin B is mainly used for pneumosporidiosis, meningeal sporotrichosis, disseminated sporotrichosis, and AIDS-combined sporotrichosis; the recommended dose is 3–5 mg/kg/d for pneumosporidiosis, disseminated sporotrichosis, and 5 mg/kg/d for meningeal sporotrichosis [36]. Azoles and potassium iodide are not recommended for pregnant women, whose treatment regimen may be localized heat therapy or medication delayed until the end of pregnancy [35]. It is important to note that for disseminated cases, the use of iodide is ineffective because it is an immunostimulant. This is also the basis for our initial treatment of choosing intravenous fluconazole and then using itraconazole instead of potassium iodide for this patient with skin dissemination [[37], [38], [39]].

This is the first systematic review of the different sporothrix species of disseminated subcutaneous sporotrichosis, but due to the small number of cases in which sporothrix species have been performed and the long time span of reported cases, there is a partial lack of information that prevents conclusions from being drawn on the association of definitive clinical types with different strains of disseminated subcutaneous sporotrichosis. Further research is needed to determine whether there is a correlation between the two.

## 4 Conclusion

Sporotrichosis is difficult to differentiate from other diseases in terms of skin lesion morphology, making fungal culture, pathological findings, and mNGS necessary. Fungal culture and pathological diagnosis are most commonly used in clinical practice; however, they also have the disadvantages of a low positivity rate and long cycle time, which may delay the optimal time for treatment. mNGS, which performs high-throughput sequencing of the genome of pathogens and can detect thousands of pathogens at a time, is more efficient and faster, but it is not fully popularized at present because of the high price of the test. In this case, the diagnosis of sporotrichosis was confirmed and clarified by the identification of *Sporothrix schenckii* based on pathological findings and mNGS, which provided guidance for the selection of antifungal drugs in the treatment process.

## CRediT authorship contribution statement

**Guangqi Zhu:** Writing – original draft. **Luwen Zhang:** Writing – original draft. **Yan Wang:** Supervision. **Zheen Zhang:** Writing – review & editing.

## Ethics approval and consent to participate

Not applicable.

## Clinical Trial

Not applicable.

## Consent for publication

Written informed consent for publication was obtained from the patient.

## Data availability statement

Not applicable.

## Funding support

Not applicable.

## Declaration of competing interests

All authors have no conflict of interest related to this publication.
